# Clinical value of combined predictors of RET%, γ-GT, LDH in the ABO neonatal hemolytic disease

**DOI:** 10.3389/fped.2023.1265739

**Published:** 2023-12-01

**Authors:** Xiaoxiao Liu, Yan Dong, Yingchao Qin, Chunyan Xue, Wei Lyu

**Affiliations:** Department of Pediatrics, Shanghai Ninth People’s Hospital, Shanghai Jiao Tong University School of Medicine, Shanghai, China

**Keywords:** combined predictor, ABO hemolytic disease, newborn, lactate dehydrogenase (LDH), γ-glutamyltransferase (γ-GT)

## Abstract

**Objective:**

The purpose of this study is to examine the prognostic significance of the amalgamated indicators, reticulocyte percentage (RET%), lactate dehydrogenase (LDH), and γ-Glutamyltransferase (γ-GT), in neonatal ABO hemolytic disease.

**Methods:**

A total of 137 hospitalized children with pathological jaundice were included. Based on their medical conditions, they were categorized into two groups, hemolytic (67 cases) and non-hemolytic (70 cases). Pearson linear correlation and binary logistic multivariate analysis were used to analyze LDH, γ-GT, RET% and hemolysis. Furthermore, the predictive value of the combined predictors of RET%, LDH, and γ-GT on ABO neonatal hemolytic disease was evaluated using the ROC curve analysis.

**Results:**

The laboratory indexes of the two groups were subject to analysis using binary logistic regression to identify suspicious influencing factors. The study revealed that RET%, LDH, and γ-GT were independent risk factors for hemolysis. Pearson linear correlation analysis indicated a positive correlation between LDH and γ-GT with RET% (*r* = 0.529, *P* < 0.01; *r* = 0.526, *P* = <0.01, respectively). Furthermore, the predictive value of each combined predictor was obtained using the ROC curve, and it was observed that combined predictor L (RET% + LDH + γ-GT)>L1 (RET% + LDH)>L2 (RET% + γ-GT).

**Conclusion:**

Combined predictor L (RET% + LDH + γ-GT)demonstrate its optimal diagnostic efficacy, offering a novel approach towards diagnosing early-onset ABO hemolytic disease of the newborn.

## Introduction

Hemolytic disease, which is caused by ABO blood type incompatibility, is a frequently occurring ailment in newborns, particularly those who are type A or B and are born to mothers with blood type O (1). Despite 10% of pregnancies with ABO blood group incompatibility resulting in neonatal hemolytic disease, ABO hemolytic disease accounts for a significant 85.3% of neonatal hemolytic disease, with an incidence rate ranging from 2.23% to 4.55% (2–4). Untimely diagnosis and delayed treatment may result in various symptoms, including jaundice, anemia, and bilirubin encephalopathy. In severe cases, it may lead to hepatosplenomegaly and even more severe heart failure, posing a severe threat to the newborn's life (5). Furthermore, reports have indicated that this ailment may harm the newborn's central system, which can significantly impact their intellectual development (6). Therefore, this ailment necessitates utmost attention.

Currently, the hemolysis three tests (direct antiglobulin test, free antibody test, and antibody release test) are the commonly employed diagnostic method (7, 8). However, this testing method is lacking in many non-pediatric specialized hospitals and grassroots hospitals. Moreover, there are few reports on the routine tests that are correlated with hemolysis. Typically, hemolysis is manifested as mild anemia with normal hemoglobin levels and without liver and spleen enlargement. However, it does lead to a certain degree of hyperbilirubinemia, which can be easily misinterpreted as physiological jaundice. Diagnosis of typical cases is uncomplicated, based on the incompatibility between the blood types of the mother and child, age, elevated bilirubin levels, and decreased hemoglobin levels. Nonetheless, it is worthwhile to explore the utilization of basic routine examinations to accurately diagnose hemolytic disease in newborns, particularly in children with mild anemia in clinical practice.

Several research studies have reported that the determination of reticulocyte (9–12) and lactate dehydrogenase percentages (13–15) is useful in the diagnosis of ABO hemolytic disease in newborns. The γ-Glutamate transferase is also present in the liver, and it has not been reported whether it is also related to the occurrence of neonatal hemolytic disease (16). The potential of combined predictive factors comprising these non-specific detection indicators for accurate diagnosis of ABO hemolytic disease in newborns warrants investigation by clinical practitioners. This study seeks to investigate the value of RET%, LDH, γ-GT, and ABO as combined predictive factors in accurately diagnosing ABO hemolytic disease in newborns, thereby providing a reference basis for clinical diagnosis.

## Materials and methods

### Population and design of the study

This retrospective case-control study was conducted in the department of pediatrics, Shanghai Ninth People's Hospital, Shanghai Jiao Tong University School of Medicine during the period of June 2015–June 2021. The study was approved by the Ethics Committee of the Shanghai Ninth People's Hospital (SH9H-2021-T404-1, November, 2021).

137 hospitalized neonates with pathologic jaundice were above 37 weeks of gestation and 2.5 kg of birth weight and day ages less than 7 days were divided into hemolytic group (67 cases) and non-hemolytic group (70 cases). The three hemolytic tests are utilized as diagnostic tools for ABO hemolytic disease in newborns. The presence of any of the following three conditions indicates a positive result for the hemolysis test: 1. Positive results for the antibody release test, direct anti-human globulin test, and free antibody test. 2. Positive results for the antibody release test and direct anti-human globulin test, but negative result for the free antibody test. 3. Positive result for either the antibody release test or direct anti-human globulin test, but negative result for the free antibody test (Table 1). Neonates are excluded if they have congenital malformation, metabolic disease, metabolic disorders caused by dehydration and sever infection, hemolytic disease resulting from Rh incompatibility, glucose 6-phosphate dehydrogenase (G6PD) deficiency, low birth weight, anoxia, asphyxia, septicemia, cephalhematoma, or polycythemia vera.

**Table 1:** Clinical characteristic of three hemolysis results.

Test items	ABO-HDN	Suspicious ABO-HDN	Non ABO-HDN
Direct antiglobulin test	+ −+−	− +	–
Free test	++− −	+ −	–
Release test	+++ +	− −	–

### Data collection

Blood routine, reticulocyte percentage, blood type, hemolysis and liver function indexes were performed for 137 hospitalized neonates on admission. The semi-automatic immune turbidimeter, Turbo X-plus kit, provided by Orion company, the XT-2000i full-automatic blood cell analyzer manufactured by Sysmex company, and the cobasintegra 400plus full-automatic biochemical analyzer manufactured by Roche company in Germany were used.

### Statistical analysis

All of the data were analyzed using an unpaired Student *t* test for continuous variables and the *χ*^2^ test or Fisher's exact test for categorical variables using SPSS version 25. Pearson linear correlation and binary logistic regression were used to analyze RET%, LDH, γ-GT on the correlation of ABO hemolytic disease of newborn. Taking hemolysis as the state variable, the predictive value of nonspecific laboratory indicators combined with predictors for hemolytic disease of newborn was analyzed by a receiver-operating characteristic (ROC) curve. *P *< 0.05 was considered statistically significant.

## Results

There were 67 neonates in hemolytic group and 70 in non-hemolytic group. There was no significant difference in gestational age, sex (male/female) and birth weight between the two groups (*P* > 0.05). Notably, significant differences were found in pregnancy times and stool times between the two groups (*P < *0.05). Additionally, the data demonstrated that the white blood cell (WBC), neutrocyte (*N*)%, eosinophilic granulocyte (EO)%, red blood cell (RBC), hemoglobin (Hb), hematocrit (HCT), mean corpuscular volume (MCV), mean corpuscular hemoglobin (MCH), mean corpuscular hemoglobin concentration (MCHC), red blood corpuscular volume distribution width (RDW-CV), red blood corpuscular volume distribution width (RDW-SD), RET, total bilirubin (TBIL), γ-GT, and LDH values were significantly different in the hemolytic group when compared to the non-hemolytic group (*P < *0.05). There was a significant difference in the hemolytic three items between the two groups (*P < *0.01), indicating that the maternal pregnancy, stool frequency, WBC, N%, EO%, RBC, Hb, HCT, MCV, MCH, MCHC, RDW-CV, RDW-SD and RET%, TBIL, γ-GT, lactate dehydrogenase (LDH) and hemolysis may be potential factors that influence hemolysis of ABO newborns. However, there was no statistical difference in MCH in blood routine between the two groups (*P = *0.264, *P > *0.05), leading to the conclusion that MCH was not a suspected influencing factor of hemolysis in ABO newborns (Table 2).

**Table 2:** Comparison of clinical data between ABO hemolytic group and non-hemolytic group.

Characteristics	ABO HDN (*n* = 67)	Non ABO-HDN (*n* = 70)	Statistics (*t*/*x*^2^)	*P*-value
Age, week, mean (SD)	39.11 ± 1.40	39.10 ± 1.08	−0.042	0.966
Birth weight, g, mean (SD)	3,325.37 ± 459.14	3,358.57 ± 443.00	−4.3	0.668
Gender, *n* (%)			2.916	0.088
Male	22 (32.84)	33 (47.14)		
Female	45 (67.16)	37 (52.86)		
Blood type, *n* (%)			3.025	0.216
A	34 (50.74)	27 (38.57)		
B	33 (49.25)	43 (61.43)		
Stool frequency, second			5.08	0.024
>5	41 (61.19)	48 (68.57)		
≤5	26 (38.81)	22 (31.43)		
Maternal pregnancies, second			8.53	0.004
>2	17 (25.37)	13 (18.57)		
≤2	50 (74.63)	57 (81.43)		
Leukocyte, 10^9^/L, mean (SD)	15.57 ± 6.05	11.22 ± 3.06	5.345	<0.05
*N*, %, mean (SD)	62.40 ± 11.22	44.12 ± 12.80	8.887	<0.05
EO, %, mean (SD)	2.86 ± 1.97	4.43 ± 2.16	−4.45	<0.05
Erythrocyte, 10^9^/L, mean (SD)	4.32 ± 0.64	4.80 ± 0.51	−4.95	<0.05
HB, g/L, mean (SD)	151.99 ± 20.98	168 ± 19.63	−4.69	<0.05
HCT, %, mean (SD)	41.78 ± 9.52	46.03 ± 7.44	−2.923	0.004
MCV, fL, mean (SD)	99.46 ± 3.97	97.08 ± 3.08	3.93	<0.05
MCH, pg, mean (SD)	35.28 ± 1.63	35.01 ± 1.24	1.121	0.264
MCHC, g/L, mean (SD)	354.06 ± 11.84	360.64 ± 9.32	−3.62	<0.05
RDW-CV, g/L, mean (SD)	17.42 ± 1.50	15.62 ± 0.84	8.59	<0.05
RDW-SD, fL, mean (SD)	62.07 ± 6.06	55.13 ± 3.45	8.19	<0.05
RET, %, mean (SD)	5.37 ± 1.82	2.08 ± 1.06	12.87	<0.05
TBIL, µM, mean (SD)	197.85 ± 90.67	259.43 ± 46.75	−4.96	<0.05
LDH, U/L, mean (SD)	595.84 ± 157.30	442.04 ± 142.43	−6.00	<0.01
γ-GT, U/L, mean (SD)	138.61 ± 77.67	105.27 ± 64.66	−2.74	<0.01
Hemolysis three, *n* (%)				
Negative	6 (8.96)	64 (91.43)	93.563	<0.01
Positive (DAT Positive/Release test Positive)	10 (14.93)	0 (0)		
Dissociation test Positive	51 (7.61)	6 (8.57)		

HDN, hemolytic disease of the newborn; DAT, the direct antiglobulin test; SD, standard deviation; WBC, leukocyte; N, neutrophils; EO, eosinophil; HB, haemoglobin; HCT, hematocrit; MCV, mean corpuscular volume; MCH, mean erythrocyte hemoglobin content; MCHC, mean erythrocyte hemoglobin concentration; RDW-CV, red blood cell volume distribution width-CV; RDW-SD, red blood cell volume distribution-SD; RET, reticulocyte; TBIL, serum total bilirubin; LDH, lactate dehydrogenase; γ-GT, γ-glutamyltransferase.

Within the ABO HDN cohort, a statistically significant difference was observed in LDH and γ-GT levels between the positive and negative release test groups across the three hemolysis tests (*P < *0.05). In contrast, no statistically significant difference was observed in LDH and γ-GT levels among the three hemolysis tests (*P* > 0.05). These findings indicate a noteworthy association between higher LDH and γ-GT levels and increased positivity rates of the release test, as evidenced by Table 3.

**Table 3:** The correlation of LDH, γ-GT and hemolysis three.

Characteristics	*n*	LDH	γ-GT	*t*	*P*-value
DAT					
Positive	45	613.22 ± 193.66	258.62 ± 181.97	1.157	0.251
Negative	22	548.27 ± 255.90	285.64 ± 218.74	−0.534	0.595
Release test					
Positive	28	669.61 ± 194.72	210.82 ± 145.09	2.598	0.012
Negative	39	536.10 ± 216.02	308.18 ± 214.58	−2.081	0.041
Dissociation test					
Positive	38	627.82 ± 184.88	249.45 ± 168.99	1.573	0.120
Negative	29	544.83 ± 247.10	291.14 ± 222.59	−0.872	0.386

**Figure 1:**
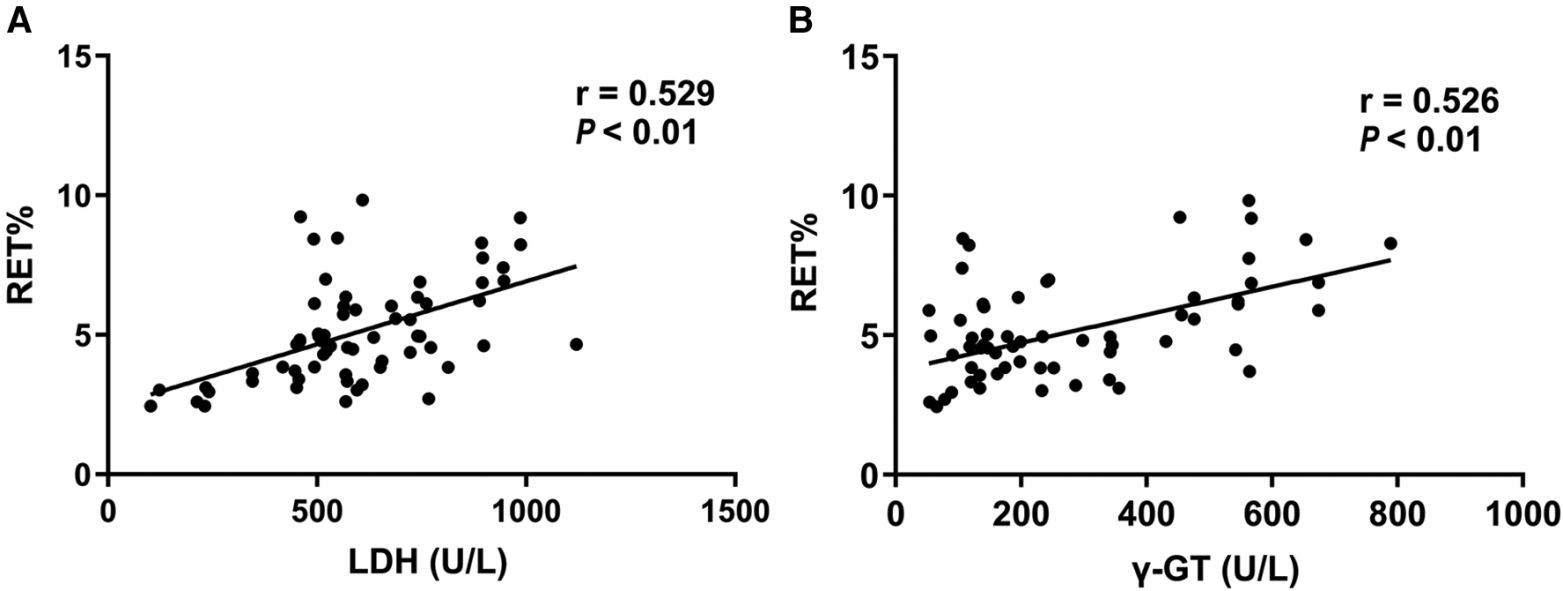
Correlation analysis among LDH, γ-GT and RET% in children with ABO hemolytic disease.

Pearson linear correlation analysis found that the level of LDH and γ-GT of 67 neonates in hemolytic group was linearly and positively correlated with RET% level (Figure 1, *r* = 0.529, *P *< 0.01; r = 0.526, *P *< 0.01).

In this study, the state variable analyzed was ABO hemolytic disease of newborn. Maternal pregnancy times, stool times, and various blood indicators such as WBC, N%, EO%, RBC, Hb, HCT, MCV, MCH, MCHC, RDW-CV, RDW-SD, RET, TBIL, γ-GT, LDH, and hemolysis were examined as independent variables. Binary logistic analysis revealed that RET%, LDH, and γ-GT were identified as independent risk factors for hemolysis (*P < *0.05). The analysis further showed that the increase in the probability of hemolysis was associated with significant increases in RET%, LDH, and γ-GT, with an OR of 7.439, 1.006, and 1.019, respectively. To obtain a combined predictor of these variables, we used the formula for calculating variables in the transformation in SPSS. The resulting formulas for the combined predictors were L = RET% + β (LDH)/β (RET%) × LDH + β (γ-GT)/β (RET%) × γ-GT, L1 = RET% + β (LDH)/β (RET%) × LDH, and L2 = RET% + β (γ-GT)/β (RET% × γ-GT (Table 4).

**Table 4:** Binary logistic multivariate analysis of hemolytic group and non-hemolytic group of ABO newborns.

Variable	Regression coefficient	Standard error	Wald	*P*-value	OR (95% CI)
RET%	2.007	0.388	26.810	0.000	7.439 (3.480–15.899)
LDH	0.006	0.002	5.630	0.018	1.006 (1.001–1.010)
γ-GT	0.019	0.007	6.895	0.009	1.019 (1.005–1.033)

**Figure 2:**
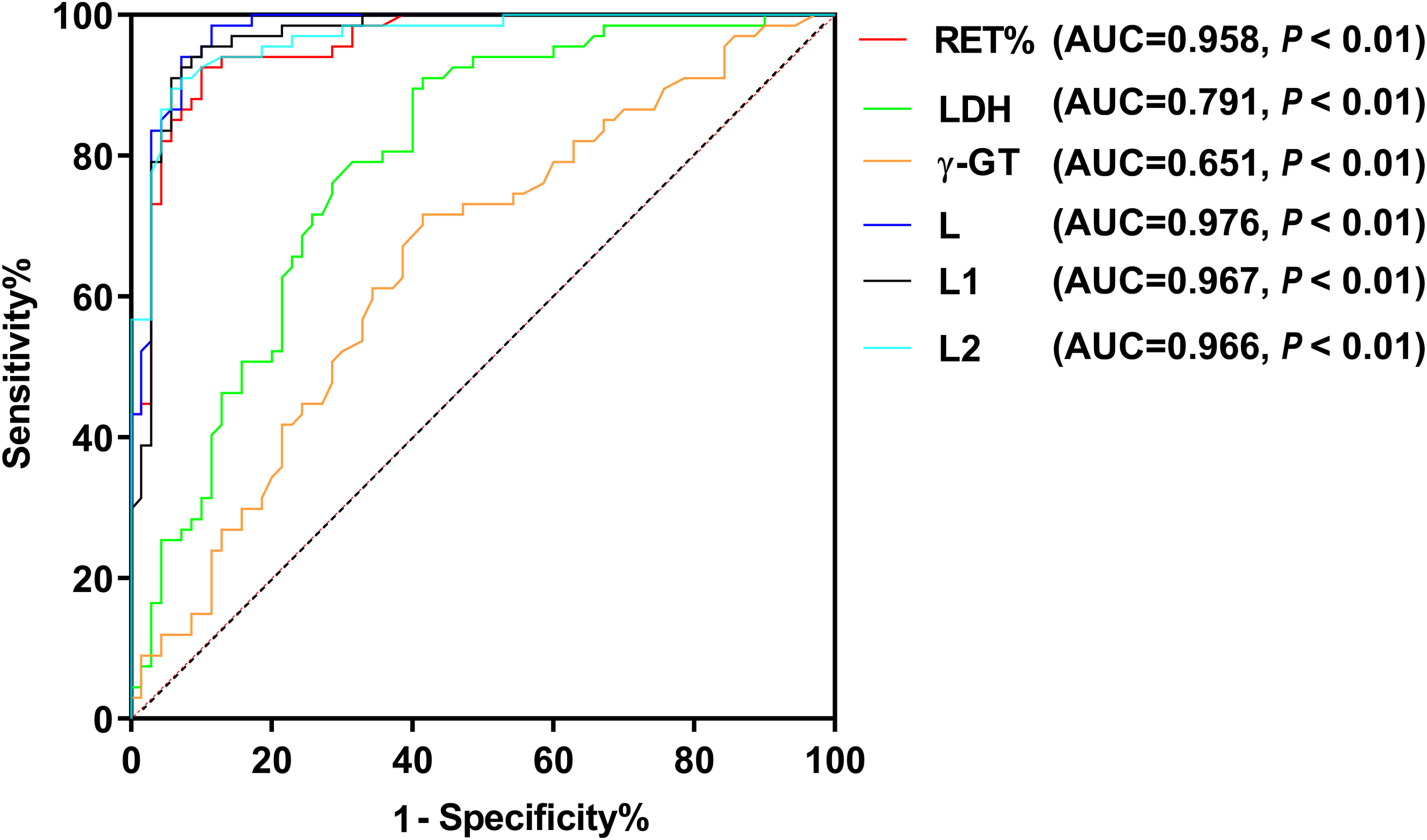
ROC curves for RET%, LDH, γ-GT and the joint predictive factors in the blood.

Based on the analysis of ABO hemolytic disease as the outcome variable, in comparison to the non-hemolytic group which served as the reference, the ROC analysis demonstrated that the AUC 95% CI of the combined predictors L, L1, L2 and RET%, LDH, γ-GT were all greater than 0.6. Additionally, the AUC 95% CI of the combined predictors L, L1, L2 were all greater than 0.9. These six variables, L > L1 > L2 > RET% > LDH > γ-GT, have significant value in predicting ABO hemolytic disease of newborn (Figure 2).

## Discussion

Within cases of ABO blood group incompatibility between mothers and infants, approximately one-fifth of cases develop ABO hemolytic disease (17). The majority of affected children exhibit no additional abnormalities beyond jaundice. In most cases, jaundice is observed on the second or third day after birth, frequently accompanied by varying degrees of anemia (18). A subset of children may experience late anemia within three to six weeks post-birth as a result of antibody persistence, which can be easily overlooked (19). Furthermore, neonatal liver function often proves insufficient in metabolizing bilirubin, leading to its accumulation in the body. Additional symptoms such as hepatosplenomegaly may also present and can result in brain stem auditory conduction pathway damage, bilirubin encephalopathy, or even death in severe cases (20, 21). Our hospital serves as a neonatal critical care center within Baoshan District, Shanghai. Diagnostic tests for ABO blood group incompatibility are often not available at primary care facilities, leading to overlooked hemoglobin drops which can be misdiagnosed as physiological jaundice. Early detection of hemolysis through non-specific laboratory indicators and prompt treatment can prevent related complications and prove to be of great significance in safeguarding the health of newborns.

Reticulocytes present in the bloodstream of neonates serve as indicators not only of the level of hematopoietic compensation in children but also of the pathological processes that arise from erythrocyte decomposition and heme metabolism during hemolysis (22–24). Extensive research supports the usefulness of reticulocytes in assisting the diagnosis of hemolytic disease in newborns (25). In a study involving the carboxyhemoglobin (COHb) and reticulocyte count of hemolytic and non-hemolytic newborns, the reticulocyte count of the hemolytic group was found to be considerably higher than that of the non-hemolytic group (26). This suggests that hemolysis may be predicted by monitoring the levels of hemoglobin and reticulocytes during the early stages of hemolysis. Furthermore, the RET% was discovered to be an independent risk factor in the incidence of ABO hemolytic disease in newborns. Correspondingly, the probability of hemolysis increased by 7.439 times for every 1% increase in RET%. Based on the ROC curve, the critical value of RET% was established to be 3.015%, with a 92.5% sensitivity and a 90% specificity. The resulting AUC area was 0.958, indicating that when RET% is above 3.015%, the occurrence of hemolysis is more likely. These findings concur with analogous research outcomes obtained both locally and abroad.

The present study retrospectively analyzed 67 infants with ABO hemolytic disease of newborn and compared them with 70 infants in the non-hemolytic group. The results showed that γ-GT levels were significantly higher in the hemolytic group (*P < *0.05). Additionally, LDH has been identified as a potential biomarker for evaluating hemolysis occurrence in children since it is widely distributed in all human tissues (27). Our study supports this notion and indicates that LDH was an independent risk factor for ABO hemolytic disease of the newborn, with a β coefficient of 0.006 > 0 or =1.006, indicating that the probability of hemolysis increases by 1.006 times for every 1 unit increase in LDH. Furthermore, the study found that γ-GT was positively correlated with neonatal serum total bilirubin, and it was identified as an independent risk factor for ABO hemolysis in neonates with a β coefficient of 0.019 > 0 or =1.019. Our study also demonstrated that the higher the γ-GT value, the higher the positive rate of the release test in the three hemolysis tests, and the observed difference was statistically significant (*P *< 0.05). These findings provide valuable insights to aid in clinical decision-making for the management of ABO hemolytic disease of the newborn.

ROC curve analysis indicates that the combination of RET%, LDH, and γ-GT in predicting hemolysis shows a significantly higher value than solely relying on ABO hemolytic disease in newborns or a combination of ABO and the mentioned predictors. Notably, these joint predictors demonstrate a greater predictive value when compared to the joint predictors of RET% + LDH (L1), RET% + γ-GT (L2), and independent predictions. Thus, it is recommended to conduct all three hemolysis tests for newborns suspected of this disease to predict the occurrence of ABO hemolytic disease comprehensively. Newborns with high suspicion of the disease should be promptly transferred to qualified superior hospitals to enhance the treatment success rate.

In conclusion, the percentages of reticulocytes, LDH and γ-GT serve as distinct prognostic indicators for early-onset ABO hemolytic disease in neonates. These biomarkers have been observed to be markedly elevated in affected neonates, and can be effectively utilized as predictors for the onset of ABO hemolytic disease during the early stages. Furthermore, the combination of these three predictors yields the most effective early prediction accuracy, highlighting its importance in the early prevention and treatment of ABO-HDN.

## Data Availability

The original contributions presented in the study are included in the article/supplementary material, further inquiries can be directed to the corresponding author.
